# Supplementary material for: Interplay between Nucleoid-Associated Proteins and Transcription Factors in Controlling Specialized Metabolism in Streptomyces
Source: mBio. 2021 Jul 27;12(4):e01077-21. doi: 10.1128/mBio.01077-21 (PMC8406272; doi:10.1128/mBio.01077-21)
Supplement: FIG S1 [file mbio.01077-21-sf001.pdf]

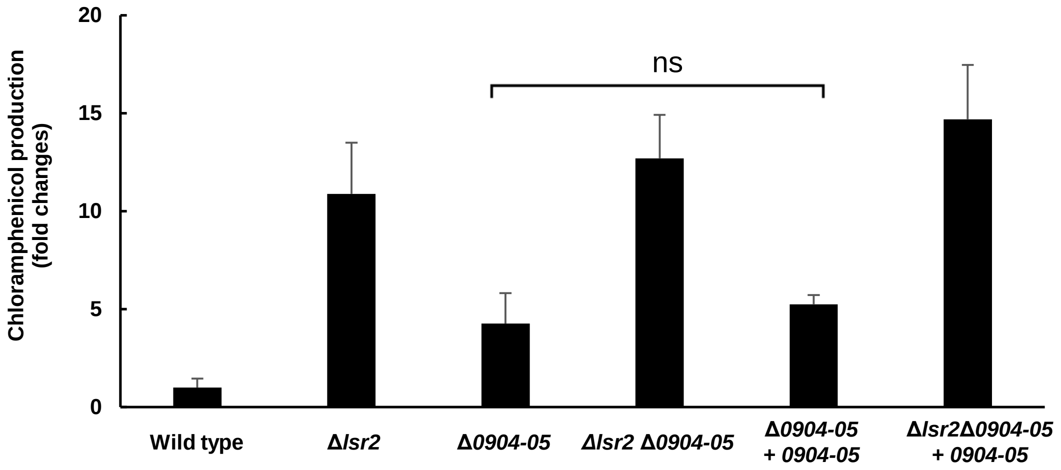

Supplementary figure 1: Complementing *sven0904-0905* failed to restore chloramphenicol production. *sven0904-0905* was deleted in wild type and Δ*lsr2* backgrounds, and the operon was re-introduced into the mutant strains on an integrating plasmid vector (+0904-05). LC-MS analyses were performed to quantify changes in chloramphenicol production, relative to empty plasmid-containing wild type and *lsr2* mutant strains. Error bars represent standard deviation for three independent biological replicates, with n.s indicating differences that were not statistically significant (comparisons with other strains were not statistically assessed).
