# Supplementary material for: Interplay between Nucleoid-Associated Proteins and Transcription Factors in Controlling Specialized Metabolism in Streptomyces
Source: mBio. 2021 Jul 27;12(4):e01077-21. doi: 10.1128/mBio.01077-21 (PMC8406272; doi:10.1128/mBio.01077-21)
Supplement: FIG S2 [file mbio.01077-21-sf002.pdf]

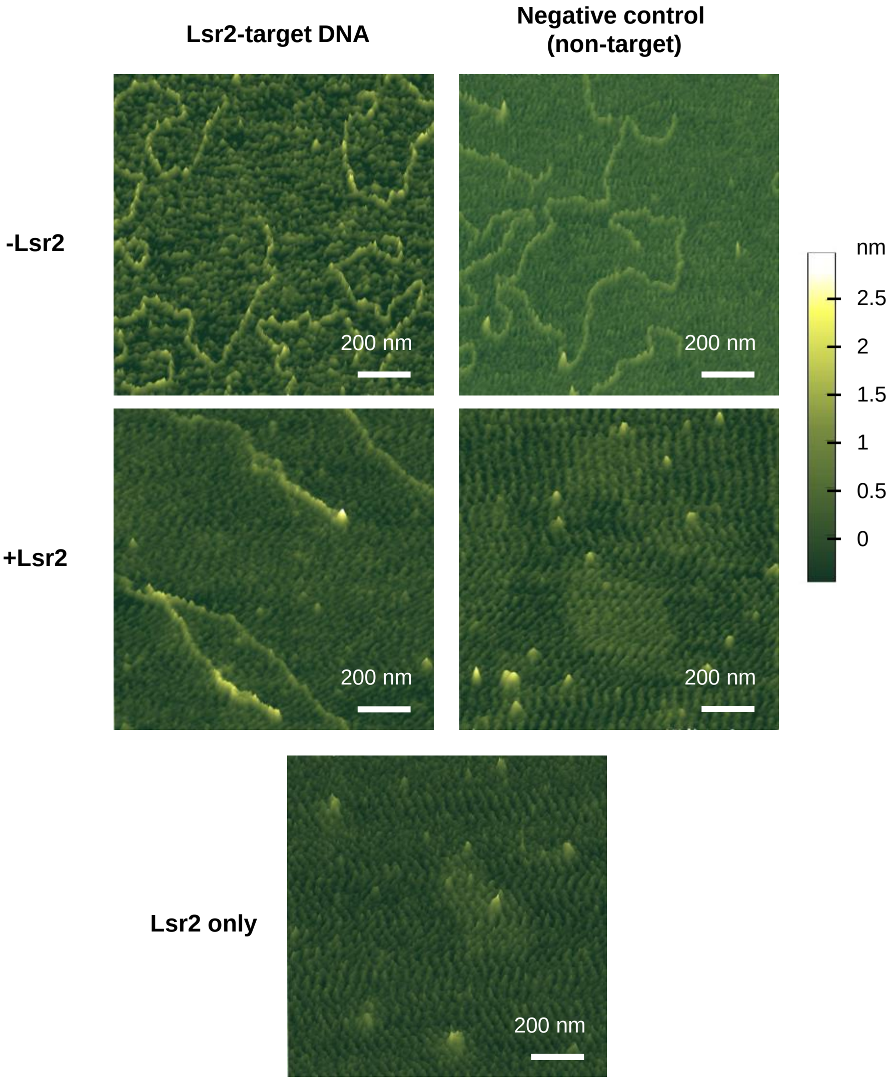

Supplementary Figure 2: Comparing Lsr2 binding to engineered target DNA (two Lsr2 binding sites) and negative control (no Lsr2 binding sites) DNA.

Top: AFM images of Lsr2 target-containing DNA (*sven0904-0905-0926*) and negative control DNA (*sven7031*) molecules with nickel added to the buffer.

Middle: AFM images of 0.5 nM DNA + 250 nM Lsr2, without nickel added to the buffer.

Bottom: AFM image of 250 nM Lsr2, without nickel added to the buffer.

The figure shows representative images from three independent experiments.
