# Supplementary material for: Interplay between Nucleoid-Associated Proteins and Transcription Factors in Controlling Specialized Metabolism in Streptomyces
Source: mBio. 2021 Jul 27;12(4):e01077-21. doi: 10.1128/mBio.01077-21 (PMC8406272; doi:10.1128/mBio.01077-21)
Supplement: FIG S3 [file mbio.01077-21-sf003.pdf]

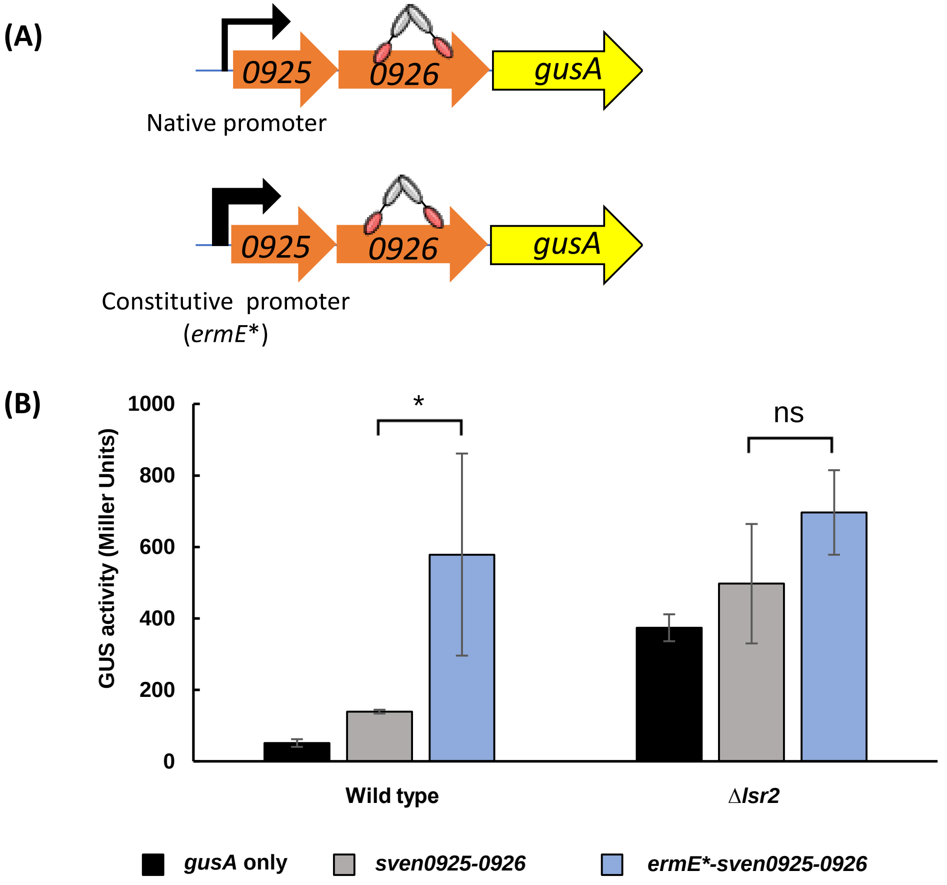

Supplementary Figure 3: Enhancing transcription overcomes the repressive effects of Lsr2. **(A)** Schematic diagram illustrating the *gus* reporter construct design. Top: The CmlR binding site, *sven0925* promoter and downstream *sven0925-0926* coding sequence was cloned upstream of the promoterless *gusA*. Bottom: a constitutive promoter, *ermE**, replaced the CmlR binding site and *sven0925* promoter, upstream of the *sven0925_0926* coding sequence in the promoterless *gusA* reporter construct. **(B)** The constructs from (A) were tested in wildtype and Δ*lsr2* backgrounds. As a negative control, the promoterless *gusA* construct was introduced into both backgrounds. Error bars represent standard deviation of the mean, for biological triplicate samples. * indicates $p<0.05$.
